# Towards a universal and privacy preserving EEG-based authentication system

**DOI:** 10.1038/s41598-022-06527-7

**Published:** 2022-02-15

**Authors:** Amir Jalaly Bidgoly, Hamed Jalaly Bidgoly, Zeynab Arezoumand

**Affiliations:** 1 Department of Information Technology and Computer Engineering, University of Qom, Qom, Iran; 2 Department of Electrical and Computer Engineering, Isfahan University of Technology, 84156-83111 Isfahan, Iran

**Keywords:** Classification and taxonomy, Computer science

## Abstract

EEG-based authentication has gained much interest in recent years. However, despite its growing appeal, there are still various challenges to their practical use, such as lack of universality, lack of privacy-preserving, and lack of ease of use. In this paper, we have tried to provide a model for EEG-based authentication by focusing on these three challenges. The proposed method, employing deep learning methods, can capture the fingerprint of the users’ EEG signals for authentication aim. It is capable of verifying any claimed identity just by having a genuine EEG fingerprint and taking a new EEG sample of the user who has claimed the identity, even those who were not observed during the training. The role of the fingerprint function is similar to the hash functions in password-based authentication and it helps preserve the user’s privacy by storing the fingerprint, rather than the raw EEG signals. Moreover, for targeting the lack of ease of use challenge, Gram-Schmidt orthogonalization process reduces the required number of channels to just three ones. The experiments show that the proposed method can reach around 98% accuracy in the authentication of completely new users with only three channels of Oz, T7, and Cz.

## Introduction

Authentication is the process of verifying a claimed identity based on the presented information or objects. With the fast growth of threats, authentication correspondingly becomes more and more vital to secure access to information and systems. Authentication can be done regarding what the user knows/has/is. *Something that the user is*, often refers to the biological aspects of the human being, namely “*biometric features*”. Current commercialized biometric features cover both physiological ones (e.g., fingerprint, face, iris, palm vein), or behavioral ones (e.g., keystroke pattern, voice, signature)^1^. Brain activity is a class of behavioral features that recently has gained much interest due to unique advantages, including its hidden but reliable nature^2,3^.

Brain activities are unique and dependent on the mental condition, performing actions, and emotional states^4^. Besides, it reveals both the behavioral and physiological features. Meanwhile, because of the state-dependent dynamic behavior and being hidden, it is naturally robust to the spoofing attacks, such as imitation/forging a user^5,6^. Nevertheless, brain activity can be simply measured by “*Electroencephalogram*” (EEG) that is placing electrodes on the scalp and recording electrical signals generated by the synaptic activation of groups of neurons.

The advantages above, attract the researchers’ interest in brain activities, especially EEG, as a potential approach for biometric purposes in recent years^7–10^. Advances in the machine learning methods, such as deep learning approaches, also accelerate this trend and lead to substantial results^11–14^. However, the current researches still suffer some major challenges such as universality, being user friendly, and preserving user privacy in the real scenarios of EEG authenticating^3^. Targeting these challenges, we propose a novel and universal EEG biometric authentication system based on the deep learning approach in this research. The contributions of this research is as follows:

- Proposed a universal EEG fingerprinting model for authentication which is capable of working on newly enrolled users (whose EEG samples are not available in the training phase) with an accuracy of 98%;
- Provided a hash function similar approach for EEG privacy-preserving that keeps users’ private information hidden by storing the EEG fingerprint, rather than the raw EEG signals;
- Reduced the required EEG channels to three channels using Gram-Schmidt orthogonalization process while maintaining accuracy up to 99.9%, which outperforms state-of-the-art works in this field;

The rest of the paper is as follows. In the next section, we will initially describe common EEG authenticating methods, and then discuss the open challenges and our motivation in more detail. In Literature Review, related prior studies are reviewed. In the following in “Proposed method” section, the schema of our proposed authentication system is introduced. Then, in “Experiments and results” section, the proposed method is evaluated, and the results are presented. Finally, the paper ends with the “Conclusion and future works”.

## Problem statement and motivation

Traditionally, an EEG biometric authentication is seen as a classification problem in that the EEG signals of a group of users are used to train a classification model as follows. Initially, users’ EEG signals are recorded while they usually silently rest or perform a specific task, such as recalling an image or mentally doing a task. After recording, the signals may be processed, and then a set of features is extracted. Afterward, the classifier is trained on the extracted features^3^. The classifier may perform either user identification or user authentication. In the former, the classifier detects the identity of a given EEG record, while in the latter, given two records of EEG, the classifier verifies that both records belong to the same user (hereafter we use term EEG biometric standing for both EEG identification and EEG authentication, although the target of this paper is EEG authenticating). It is important to note in order for the classifier to work properly, users should repeat the same task done during the initial recording.

Traditionally, shallow classifiers such as “*Support Vector Machine*” have been used for EEG biometric, but in recent years many of them are dominated by deep learning approaches, such as *convolutional neural networks*. Although deep learning classifiers have reached very high accuracy in EEG biometric, they still suffer “*Universality requirement*” which means that a biometric factor should be able to be used by everyone, not just a group of users^3,15^. The current state-of-the-art methods in EEG biometric, due to the nature of classifiers, are only able to work properly on users who have been visited during training. Consequently, if a new user is going to register in the system, his/her EEG record should be added to the training data set and the classifier has to be re-trained from scratch. Such approaches are not applicable in practice and can only be used for systems with a limited and predefined set of users. As far as we know, there are few works, especially based on deep learning methods, for EEG biometric that can work without re-training the model for the new users. Wang et al.^16^ proposed a transfer learning method to tune an existing model to accommodate new users which obviously, it is not practical on large scale. Schons et al.^17^ have tried to propose an EEG feature extractor model using a CNN, although they did not employ the feature vectors directly, and used it as the input of a fully connected neural network which ultimately makes their approach similar to a classification model. We believe that universality is the most important challenge that requires to be considered before the practical application of EEG biometric.

Another challenge in the proposed methods is the lack of attention to the user’s privacy^3^. In the EEG authentication methods, the model receives two EEG samples and verifies whether the samples belong to one person or not. This process may require that an EEG sample of each registered user be stored in the system’s database. However, the stored EEG data can reveal some information such as sex, age, illnesses, activity, or addiction of the user^18,19^. Therefore, any method provided for EEG-based authentication should use an approach similar to the password-based authentication^3^. In the authenticating by password, the server never stores the password itself but its hash. The hash does not reveal any information about the password; however, it can still be used to verify the user. None of the currently proposed methods of EEG biometric has such a capability.

Last challenge, but not least one, is the number of electrodes required in the recent methods to achieve high accuracy, especially deep learning ones^20^. On average, 33 electrodes have been used in research^3^, which is much more than the number of available electrodes in most commercial EEG headsets. In many of these methods, it is even assumed that the user’s EEG was sampled with 64 channels which are only available by advanced medical devices. The high number of electrodes practically causes difficulties in signal gathering and reduces user satisfaction. Therefore, any practical authentication method should be able to work with a limited number of electrodes.

In this paper, we have tried to focus on these three challenges to make EEG biometric more practical, and to provide a method that not only is able to keep the accuracy and efficiency of the state-of-the-art methods, but also be able to authenticate the users who do not exist in the learning process, reduce the number of required channels to the extent of the ability of commercial devices, and protect users’ privacy by not storing the EEG records themselves.

## Authentication model

Generally, an authentication system can be defined as a tuple of (*A*, *S*, *f*, *l*) where *A* denotes authentication information, *S* is the set of stored information which is required to verify authentication information, $f:A\rightarrow {S}$ is the complementation function that generates set of *S* from authentication information set *A*, and finally $l:(A\times S)\rightarrow {\{true,false\}}$ is the authentication function that verifies an identity by a single given authentication input $a\in A$, and the stored information of claimed identity $s \in S$. As an instance, in a password-based authentication, *s* is a single password, *A* is the set of allowed passwords, *S* is the set of hashed passwords, *f* is the hash function which creates the hashes of the passwords, and *l* is the equality operator. In EEG-based authentication. *A* denotes the set of EEG sample records. *Privacy preserving* and *Universality* of an EEG-based authentication can be formally defined as follows:

- **Privacy Preserving**: The EEG signals can be used to extract information such as the user’s illness or emotion, and therefore disclosing them violates the user’s privacy. Thus it should not be kept in the system. Formally, this means that complementation function *f* is defined in such a way that it does not reveal the original information of the record. This function must be irreversible (one-way function) and cannot be used to retrieve input value *a* from an output value *f*(*a*) where $a\in A$ is a single authentication data taken from the user. Note that the system should only store set of *S*, not set of *A*.
- **Universality**: The universality indicates that the authentication system should properly work for any subject. Formally this means the *f* and *l* functions should be able to work any possible set of *A*, without the need to change the method or retrain. In particular, it should work on the subjects who are not available during the training method. As mentioned before, the current EEG-based authentication methods are limited to the subjects present during the design and training of the method.

## Literature review

Many works have been proposed in the papers on the EEG biometric methods so far, including both shallow and deep methods; though, the shallow classifiers are more common in literature^3^.

To use shallow models, it is necessary to initially extract distinguishing features of the signals, and then the model can be trained on them. In these works, the choice of the feature extraction method is very effective on the accuracy of the model. Methods such as *Autoregressive Model*^7^, *Power Spectral Density*^10^, *Wavelet Transform*^8,9^, and *Fourier transform*^21^ are some commonly used EEG feature extractor methods to name. After extracting the features, the user identification/authentication is made either by a distance-based approach or a classification method. Gui et al.^22^ and Maiorana et al.^7^, for instances, proposed EEG biometric methods based on the *Euclidean* and *cosine distances* measuring, respectively. The use of *Support Vector Machine* is also very common in papers^8,10,23^. *Artificial neural networks*^9^, *hidden Markov models*^8^, are other models used in papers.

With the advancement of deep learning methods, these methods have quickly found their place in EEG biometric and it has shown that they can achieve higher accuracy. The important advantage of these methods is that the features are not required to extract explicitly, but the model itself has the ability to recognize the features hidden in the raw data. Most of the works which have proposed a deep learning approach on EEG biometric are based on “*Convolutional Neural Networks*” (CNN) models. Das et al.^11^, for example, proposed a CNN model with four convolutional layers and two max-pooling layers which can reach $99.3\%$ accuracy in EEG-based user identification. Wu^24^ similarly reached the precision of $97\%$. Schons et al.^17^ also used data augmentation by creating overlapped time windows which led to a significant increase in the accuracy. Zhang^12^ proposed an adversarial convolutional network for the EEG biometric, which can distinguish individual characteristics from the signals recorded during different sessions. Using both global spatial and local temporal filters, Chen et al.^13^ proposed a model named GSLT-CNN. There are several other works with similar approaches. In addition to CNN models, several works have investigated the usage of other deep learning models. By combining CNNs and “*Long Short-Term Memory*” (LSTM), Sun et al.^14^ proposed a model named *1D-Convolutional-LSTM* and show that the proposed model could increase the accuracy over a pure CNN model. Wilaiprasitporn et al.^25^ have also reviewed the combination of CNN and “*Recursive Neural Networks*” families including LSTM and “*Gated Recurrent Unit*” (GRU). They concluded that GRUs are faster to train and require fewer data to generalize, even though they have comparable performance and accuracy.

## Proposed method

### System overview

**Figure 1:**
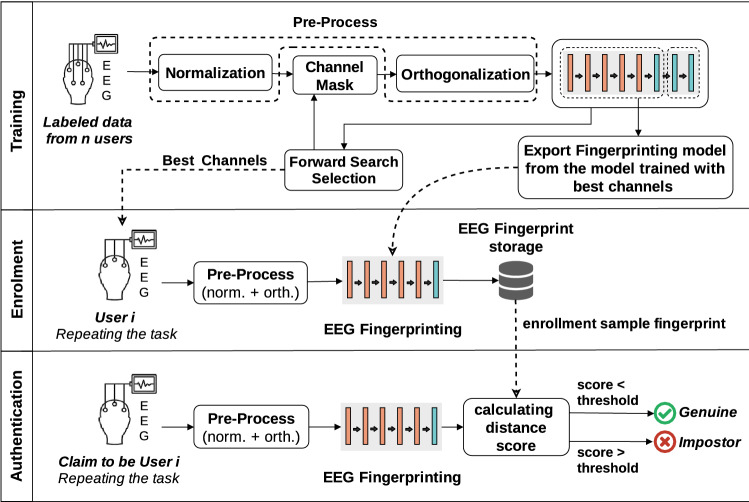
Overall schema of the proposed EEG authentication method.

Figure 1 shows the overall schema of the proposed method for the EEG authentication system. The core of this authenticator consists of a “Deep Neural Network” (DNN) based feature extractor (hereafter called EEG “*fingerprinting*”) model which is derived from a classification model. To register any user, the EEG fingerprint is extracted by this model and stored into the authentication system during the enrollment phase. To verify a claimed identity during the authentication phase, the EEG fingerprint of who tries to log in is extracted again by the model, and compared to the stored fingerprint vector of the claimed identity in the system. If two vectors are similar enough, the access is approved; otherwise, it is rejected. As it has already been mentioned in “Problem statement and motivation” section, in both phases, the user should perform the same task.

The EEG fingerprinting model is built upon a deep classification model. In the deep learning approaches, some layers of a classification model can be separated and used as a feature extractor model. Owing to this, a *n*-class classification model, which is indeed an identification system, is designed and trained on a dataset with *n* subjects in the training phase. Then, the last layers are removed to achieve a feature extractor model that indeed is our EEG fingerprinting model. The superiority of this fingerprinting model is that it can extract the feature vector of any user, even those who are not visited during the training phase. The combination of this capability with similarity measuring tools provides this opportunity to acquire a universal authentication system, without requiring to re-train the model whenever a new user registers. This approach can solve two first challenges that are universality and user privacy; see “Authentication model” subsection for more detail. To address the third challenge, a forward selection search is performed to find the most effective EEG channels, and consequently to achieve simplicity and more user-friendly during EEG recording. It is worth mentioning that channel search is done before removing the last layers of the classification model.

In the following, the classification model, effective channels’ search procedure, and the authentication mechanism are explained in more detail.

**Figure 2:**
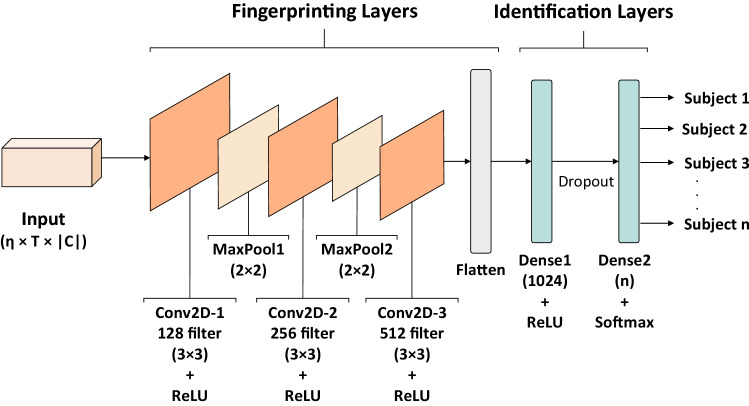
Layers of the proposed CNN identification model.

The first step of obtaining a fingerprinting model is to achieve a high accurate classification model. The deep classification model used in this paper is based on CNN models. CNN has been already used in many EEG-based authentication papers, and its capability for EEG signals classification has been well proved; see Literature Review. As it was mentioned in the system overview, the layers of the model can be split into: 1) the fingerprinting layers which are responsible for extracting features, and 2) the identification layers which identify the class of input by considering the extracted features. Here, in particular, the former consists of a sequence of convolution layers that receives a 3 dimensional matrix of EEG signal as the input and returns a matrix of features as the output. The latter consists of two fully connected layers that receive the extracted features and predict the class of the user as the output. The activation function of all layers is *ReLU* but the last layer which uses *SoftMax*. Moreover, *Maxpooling* is used as the down-sampling technique in the fingerprinting layers to reduce the model’s parameters and also to prevent over-fitting. The layers and structure of the proposed model are presented in Fig. 2. The whole model, including both fingerprinting and identification layers, is trained in an end-to-end approach; however, the identification layers will be removed from the model after the training; see “Authentication model” subsection.

**Figure 3:**
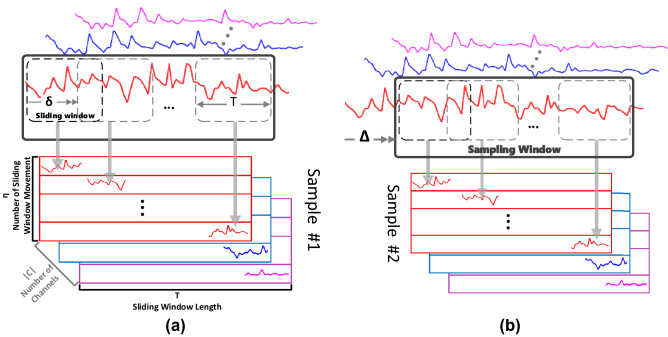
Sampling and sliding window mechanisms to generate input data of CNN from EEG time-series signals.

To convert raw EEG signals into model’s input, each channel signal is normalized using a *Min-Max scaling* method:

1 $$\begin{aligned} {\widehat{u}}_k \overset{\Delta }{=} \frac{u_k- u_k^{min}}{u_k^{max}- u_k^{min}}, \end{aligned}$$

where *u* and ${\widehat{u}}$ are raw and normalized signals, respectively, and $u_k^{max/min}$ are maximum/minimum values of the raw signals of *k*-th channel. In the following, a *sliding window* with a length of *T* is used to draw out $\eta$ overlapped segments of each EEG channel. For each segment, the sliding window moves $\delta$ units forward over the signal, and draws out a *T* length sample of the signal. The sliding step should be smaller than the window length itself (i.e. $\delta < T$); so that the extracted segments will have overlap with each other. These segments fill rows of a $\eta \times T$ matrix. For constructing the whole input matrix, this sampling procedure repeats on each channel, and creates a matrix of dimensions $\eta \times T\times |C|$ where *C* is set of selected channels. The sliding window mechanism is demonstrated in Fig. 3a. This mechanism can be utilized with any raw, normalized, or orthogonalized signal.

Considering the sliding window mechanism, the length of an EEG signal that is seen in each input is $\Gamma \overset{\Delta }{=}(\eta -1) \times \delta + T$. To generate all input data, a *sampling window* is defined with the length of $\Gamma$. For each input, this window moves $\Delta$ units forward and the whole above procedure repeats on the $\Gamma$ samples within the window; see Fig. 3b. Again, for $\Delta <\Gamma$, the generated input data will overlap each other. The sampling window mechanism results in $\frac{len(EEG)-\Gamma }{\Delta }+1$ different inputs where *len*(*EEG*) is the total length of EEG record. On the other hand, training a deep model requires a large number of inputs for each user, while the samples in available EEG datasets are usually not enough to achieve the required accuracy in this work. Thus to overcome the data shortage challenge, we consider $\Delta<< \Gamma$ to augment enough input data. 
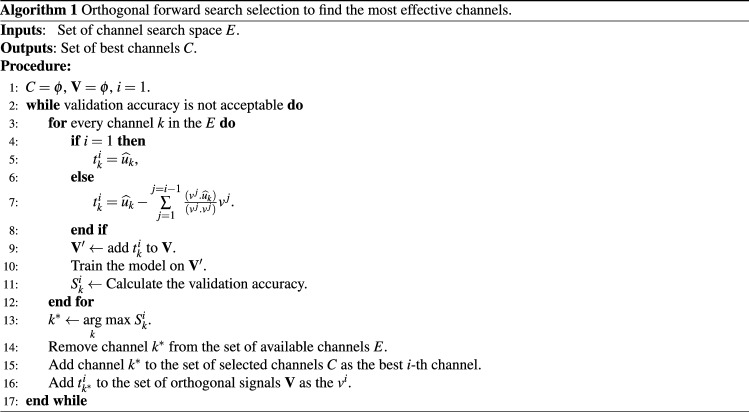


### Channel reduction

In EEG authentication, using more channels usually leads to more accuracy. However, each channel records electrical potential activities in a neighborhood and so, the nearby channels overlap and there is a redundancy between them^26^. Thus, with some compromising, we can reduce the number of the required channels while trying to keep a high accurate authentication. This also reduces the cost and complexity of EEG recording and makes the proposed system more practical. To do this, we employ a *forward search selection approach*. This approach starts with an empty set of selected channels, denoted by *C*, and the set of all channels consider as search space, denoted by *E*. Then in each step, the best channel from among the available ones is found, added to the group *C*, and removed from the search space *E*. To be sure that by adding a new channel, the new set of selected channels is still optimal, the overlapping between channels is removed by “*Gram-Schmidt orthogonalization process*” in each step as follows. Consider that ${\widehat{u}}_k$ is the normalized signal of *k*-th channel of set *E*, and $\mathbf {V}=\{v_1, \hdots , v_{i-1}\}$ is the set of the orthogonalized signals of best channels selected in order in $i-1$ previous steps of forward search. Then $v^i_k$, computed as follows, is also orthogonal to set $\mathbf {V}$:

2 $$\begin{aligned} v_k^i\overset{\Delta }{=}{\widehat{u}}_k -\sum _{j=1}^{j=i-1} \frac{(v^j.{\widehat{u}}_k)}{(v^j.v^j)}v^j, \end{aligned}$$

where (.) is the dot product, *v* is the orthogonalized EEG signal, ${\widehat{u}}$ is the normalized EEG signal. The superscript index *i* indicates the step number of forward selection approach, and subscript index *k* refers to the EEG channel. Moreover, superscript index *i* (and *j* in the sum) refers to the *i*-th (*j*-th) selected channel in the forward search selection procedure. Obviously, at the first step, i.e. $i=1$, the selected channel will not be orthogonalized. The overall orthogonal forward search selection is shown in Algorithm 1. It is worth mentioning that although this procedure can orthogonalize both raw and normalized signals, we defined the orthogonalization formula based on the normalized one due to the sequence of data pre-processing; see Fig. 1. Besides, the results of optimal channel selection are dependent on the defined task^3,27^.

The step-wise nature of the Gram-Schmidt method is completely compatible with the forward selection approach; that is at each search step, the remaining channels are initially orthogonalized to the selected orthogonal channels and then, the next optimal channel is selected. This approach not only reduces the number of required channels but also helps to achieve a high accuracy through removing the overlapped data between channels. We will show later that the accuracy of the proposed method with only 3 channels is almost close to the deep learning models used by more than 32 channels. As far as we know, the proposed method in this paper requires the lowest number of channels among other deep learning-based EEG authentication methods. In “Channel reduction results” subsection, the experimental results of the search procedure with and without orthogonalization are presented.

### EEG-based authentication

Given the trained classification model on the reduced channels set *C*, the model can now be used for EEG-based authentication system. As it was already mentioned, the layers of a classification model can be separated into two sets of fingerprinting layers and identification layers. By removing the identification layers, we achieve a fingerprinting model that is the core of our proposed authentication system. This model can extract the EEG fingerprint of any user. Now, just by comparing two fingerprints, we can determine whatever they belong to the same person or not. As mentioned before, an authentication system formally is a tuple of (*A*, *S*, *f*, *l*). Accordingly, in the proposed method, authentication information (i.e. *A*) is the set of users’ EEG samples, and the fingerprinting layers of the deep model play the role of *f* in such a case that $s=fingerprint(a)$ where *a* is a single EEG sample, and *l* is defined using the standard vectors’ distance functions. These functions are described with more details in continue.

In the user enrolment, as presented in Fig. 1, an EEG sample will be taken from the user as a single authentication data *a*, and the output of the fingerprinting model will be stored as *s*, the required information to verify the identity of the user. The fingerprinting model here plays the role of hash function in traditional password-based authentication systems. The fingerprint reveals quantitative features extracted from the EEG sample, thus similar EEG signals are expected to have similar vectors which can be indicated using any vector-based distance measuring function such as Euclidean or Cosine distance. The EEG fingerprint vector is also referred to as a *EEG hash* since it has a constant length and is irreversible similar to the hash function. Intuitively, due to the fact that the co-domain (output) size of the fingerprinting model is smaller than its domain (input) size, and the existence of non-bijection functions in the model (i.e. ReLu functions), the fingerprinting model is not an invertible function and can be considered a one-way function that keeps authentication information hidden. This is important in two ways. First, in the event of any intrusion into the authentication system, the intruder will not access users’ authentication information and thus, the security of the system will be maintained. Second, the authentication information may disclose users’ private information, so it should not be kept in plain format. The fingerprinting model proposed here not only eliminates the need to directly store the user’s EEG signals, but also by considering the fact that it is a one-way function, eliminates the possibility of disclosing the user’s private information by accessing his/her EEG signals. Therefore, it is a tool for protecting users’ privacy in EEG-based authentication. This is an advantage that has not been considered in previous studies.

To authenticate, another sample of the user is received and will be given to the function *l* along with the stored information *s*. If this function returns *true*, the identity of the claimant is verified as genuine and otherwise it is rejected as the impostor. Unlike conventional authentication systems, in EEG-based authentication the function *l* cannot be an equality operator, since the samples taken from a user cannot be exactly equal in different sessions. In the proposed method, this function is defined using the distance measurement tool as follows:

3 $$\begin{aligned} l(a,s)= {\left\{ \begin{array}{ll} true &{} distance(s,f(a)) \le threshold, \\ false &{} otherwise, \end{array}\right. }, \end{aligned}$$

where *distance* and *threshold* are the distance measurement tool and corresponding acceptable threshold, respectively, and *f* denotes the fingerprinting part of the classification model. Note that *l* does not require the EEG record itself but its fingerprint, as described in Fig. 1. However, in order to comply with the formal authentication model of “Authentication model” section, the input of the function is considered as raw EEG record (i.e. *a*) and the calculation of the fingerprint is performed inside the function by the *f* function itself. Different vector-based distance measurements can be used here, including *Euclidean*, *cosine*, or *Manhattan* distance defined as follows:

$$\begin{aligned} \left\{ \begin{array}{ll} {\text {Euclidean distance:}}&{} {dist(A,B)= \Vert A-B\Vert _2= \sqrt{\sum _{i=1}^m (a_i-b_i)^2}},\\ {\text {Cosine distance:}}&{} {dist(A,B)=\dfrac{ A \cdot B}{\Vert A\Vert _2\, \Vert B\Vert _2} = \dfrac{ \sum _{i=1}^{m}{a_i b_i} }{ \sqrt{\sum _{i=1}^{m}{a_i^2}} \sqrt{\sum _{i=1}^{m}{b_i^2}} }},\\ {\text {Manhattan distance:}}&{} {dist(A,B)= \Vert A-B\Vert _1=\sum _{i=1}^m |a_i-b_i|}, \end{array}\right. \end{aligned}$$

where $A \in \mathbb {R}^m$ and $B \in \mathbb {R}^m$ are vectors, and $\Vert \;\Vert _r$ is r-norm.

The results of studies in the experimental section show that the best choice in the proposed method is the cosine distance. Moreover, for tuning the parameter *threshold*, “*Receiver Operating Characteristic*” (ROC) or “*Detection Error Trade-off*” (DET) plots may be used. The optimal value of *threshold* is the value that minimizes both “*false acceptance rate*” (FAR) and “*false rejection rate*” (FRR) to the lowest feasible value which indeed is the bottom left corner of the DET curve. Note that in the authentication system, FAR indicates falsely accepting an imposter (Type I error), and FRR indicates falsely rejecting a genuine (Type II error) which both errors are unfavorable and should be minimized.

In practice, in order to implement this system, it is necessary to record a sample of the user’s EEG signal from the selected channels in the pre-defined conditions (such as closed/open eyes or quiet environment) during the registration stage. The system then generates the fingerprint of this input using the trained deep neural network model. Note that before feeding the EEG sample to the network, it should be normalized, orthogonalized, and augmented to generate an appropriate input with the same procedure previously described in “Proposed method” section. This fingerprint is registered in the database as the user’s stored authentication information. Whenever the user tries to log in, the system re-records the user’s EEG signal in the same pre-defined conditions, and sends it to the authentication server with the claimed identity. The server first reproduces the fingerprint from the received EEG signals and then retrieves the stored authentication information from the database based on the user’s claimed identity. It then calculates the similarity between the generated fingerprint vector and the previously saved fingerprint vector by means of cosine distance. If their distance is less than the defined threshold, the user will be authenticated and allowed to log in; otherwise, he is rejected.

### Ethical approval

This paper does not contain any studies with human or animals participants performed by any of the authors.

## Experiments and results

### Dataset and classification model tuning

In this work, we have used *Physionet EEG Motor Movement/Imagery dataset*^28,29^ to evaluate the performance and efficiency of the proposed model. The dataset is a well-known and frequently used one in EEG research and includes EEG records of 109 healthy volunteers sampled using 64 channels at the rate of 160 Hz. The position of the electrodes on the user’s scalp is an international 10-10 system. EEG signals were recorded during 14 experiments from each subject. It includes two one-minute baseline (idle) runs, one in “*open-eyed*” (REO), and another in “*closed-eyed*” (REC), and three two-minute runs with four different motor/imagery tasks.

All experiments in this section are implemented using “*Python*” v3.8, “*Keras*” v2.4^30^, and “*MNE*” v0.24^31^. In the experiments, just the REO EEG records are used which is already shown that can achieve higher accuracy in subject identification^32,33^. As it has been mentioned before, the records are first normalized and then split into input data for the model using the sliding window with $T=160$ (i.e. one-second segment of EEG signal ) and $\delta =4$. The samples are also augmented using the sampling window with $\Delta =8$ (see “Deep classification model”), so that in the end, the number of input data per subject reaches 1171.

**Figure 4:**
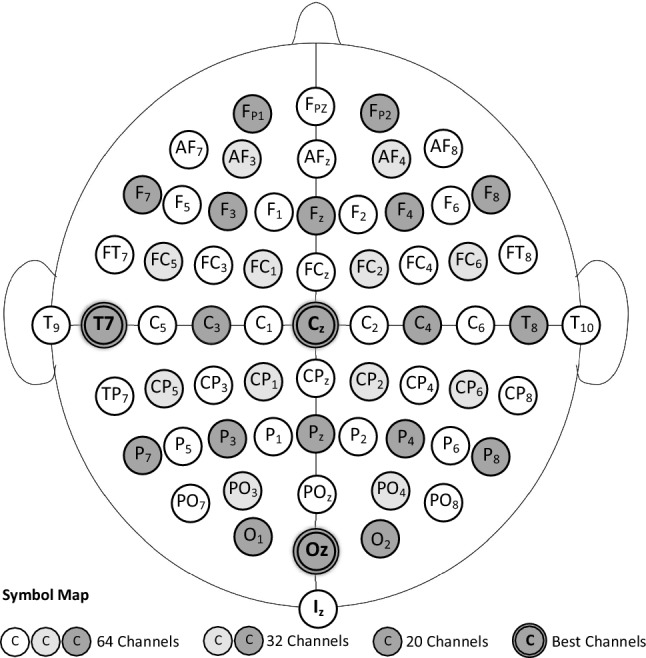
Position of all 64 electrodes on the scalp. Selected 32 channels are indicated by the color circles (both dark and light gray ones). Dark gray circles distinguish 20 channels of search space.

To set up the classification model, the model was first trained on all 64 channels and also in another experiment on only half of the channel, 32 channels (the selected channels are shown in Fig. 4). In both cases, the model’s hyper-parameters were tuned to reach the best values, so that in the end the model reached $100\%$ accuracy in the test data. The values of hyper-parameters are shown in Table 1. Moreover, the results obtained from the proposed model compared to other state-of-the-art works in EEG-based identification are shown in Table 2. As it can be seen, the proposed model performs better than the previous works, even with 32 channels and a total of 109 subjects, and achieves the highest possible accuracy.

**Table 1:** Parameters and hyper-parameters of the classification model.

Parameter	Value	Parameter	Value
$\delta$	4	$\Delta$	8
*T*	160	$\eta$	20
$\Gamma$	236	*n*	109
Learning rate	0.0001	Dropout rate	0.25
No. of epochs	30	Batch size	64
Task duration	60s	Stride	(1, 1)
Optimizer	RMSprop		

### Channel reduction results

As it was mentioned in “Problem statement and motivation” section, an important step to make the EEG authentication more practical is reducing the number of the required channels. To do this, we apply the procedure in Algorithm 1 on the model introduced in the previous subsection. Here, the search space *E* is limited to only 20 channels that are more common in the commercial EEG headsets, e.g. *Emotive Epoc*; see Fig. 4. The results of the forward search selection are shown in Table 3. To illustrate the role of orthogonalization, the search procedure is done in both cases of with and without orthogonalization for the second and third channels. At the third channel, the search procedure was stopped because of achieving an acceptable accuracy.

As we expected, the orthogonalization reduces the redundancy and reveals more insignificant features. Consequently, it improves performance in comparison to the simple search without orthogonalization. Also, by comparing the results of Table 3 with Table 2, it can be seen that our proposed CNN model that is trained with the three most effective channels, has acceptable performance and is near to the other classification models with a higher number of channels, and better performance than the models with a low number of channels. The best channels found here are also consistent with other research with the similar task^22,27^.

**Table 2:** Comparison with some state-of-the-art EEG based identification systems.

Type	Paper	Subjects	Channels	Accuracy (%)	Architecture/classifier	No. of layers
Deep	Proposed	109	64	100	CNN+Dense	8
	Proposed	109	32	100	CNN+Dense	8
	Sun^14^	109	64	99.58	CNN+LSTM+Dense	10
	Sun^14^	109	32	99.50	CNN+LSTM+Dense	10
	Sun^14^	109	4	94.34	CNN+LSTM+Dense	10
	Wang^16^	109	64	99.97	Graph CNN+Dense	6
	Wilaiprasitporn^25^	32	5	99.10	CNN+LSTM+Dense	8
Shallow	Singh^26^	109	64	100	KNN	–
	Kaur^32^	109	64	98.16	SVM, Random forest	–
	Fraschini^34^	109	64	96.9	Euclidean distance	–

**Table 3:** Best Channel selected in each step of the forward search selection with (gray rows) and without (white rows) the orthogonalization.

No. of channel(s) (step of forward search selection)	Previously selected channel (s)	Orthogonalized	Next channel	Accuracy (%)
1 ($i=1$)	$\{\}$	–	Oz	99.18
	$\{Oz\}$	$\times$	Fz	99.68
2 ($i=2$)	$\{Oz\}$	$\checkmark$	T7	99.88
	$\{Oz,\,Fz\}$	$\times$	O1	99.85
3 ($i=3$)	$\{Oz,\,T7\}$	$\checkmark$	Cz	99.92

### Authentication results

In this subsection, the authentication results are presented and discussed. For this purpose, as well as testing the authentication model, subjects in the dataset are first divided into two completely separate groups. The first group, named “*Alpha*” users, includes the first 90 subjects in the dataset, and is used to train and test the classification model. The second group, which is so-called “*Beta*” users, includes the rest (i.e., 19) of the subjects and represents the users who are going to register in the system after deploying the system. This group is used to test the performance of the model in authenticating newly enrolled users in the system; hence, no samples of their EEG signals should be seen during training or constructing the fingerprinting model. Hereafter, the fingerprinting model is created using the samples of the Alpha users recorded from three channels of Oz, T7, and Cz. The Alpha group itself is divided into two sets of training and testing data which is used to create the fingerprinting model and tune the threshold value, respectively. In these sets, the samples of all 90 subjects are randomly distributed.

**Figure 5:**
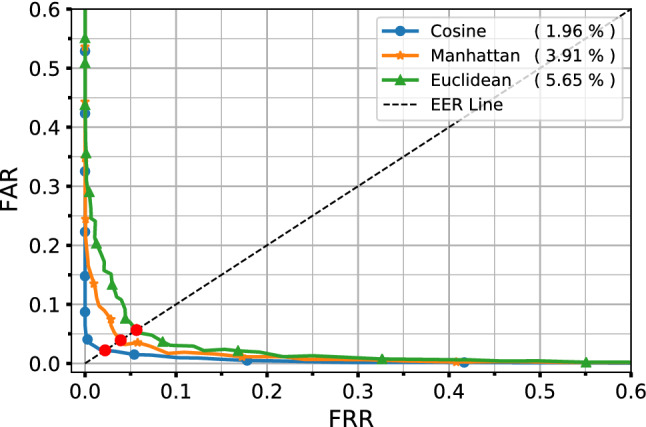
DET plot of different distance measures. The values in the parentheses are EER of corresponding distance in percent.

As it was mentioned before, authentication is done by capturing the fingerprint of the genuine identity and comparing its distance with the claimed identity. If the distance between these fingerprints is less than a pre-defined threshold, the identity will be confirmed; otherwise, the identity will not be verified. For this aim, first, the classification model is trained using the best selected orthogonalized channels (named in the previous section) on the training data of Alpha users, and the fingerprinting model is extracted from it. Then test data is used to adjust the threshold value. The threshold directly affects the performance of the authentication model. Its low value increases FRR, and its high value increases FAR (type I&II errors). According to Authentication Model, DET plot is used to adjust the threshold value. This plot shows how the errors can be traded off against each other. The optimal value of the threshold is obtained from the so called “*Equal Error Rate*” (EER) point in which the first and second types of errors are equal (or close) to each other. This point is the last point of the bottom left corner of the plot. Figure 5 shows the DET plot for different distance functions. For instance, the optimal threshold value for cosine distance is 0.275 in which both FAR and FRR are approximately equal to $98.04\%$.

In addition to the threshold value, the distance measure itself also affects the accuracy of the proposed model. To find out the best distance measure, some important measures were examined here, and their results are shown in Table 4. This table shows the results of Euclidean, Manhattan and cosine distance. In each case, the best value of the dedicated threshold for that measure is obtained in the same way as discussed before (according to Fig. 5), and is displayed in the *threshold* column of Table 4. As it can be seen, the cosine distance has the best performance and improves the accuracy compared with Manhattan and Euclidean distances, by $2\%$, and $3\%$, respectively.

**Table 4:** Comparison of Euclidean, Manhattan and cosine distances.

Distance	Threshold	EER (%)	Accuracy (%)	Precision (%)	Recall (%)
Euclidean	62.5	5.65	94.27	94.30	94.25
Manhattan	1112.5	3.91	95.80	95.52	96.10
Cosine	0.275	1.96	98.04	97.45	98.66

**Figure 6:**
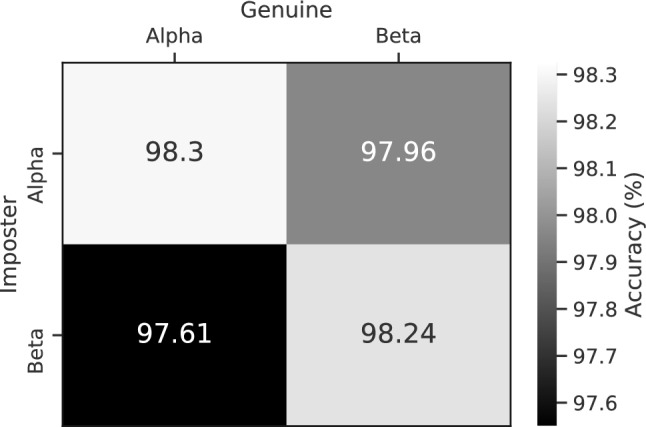
Authentication model’s accuracy in four possible cases with cosine distance. Alpha and Beta labels indicate whatever the data was used in the training and testing phase or not.

The proposed model with the best distance measure and the corresponding threshold value achieved $98\%$ accuracy in EEG-based authentication, which is outstanding and distinct from the previous works since they didn’t test their proposed method on Beta users. However, to have a more detailed assessment and identify cases in which the model has weaknesses or strengths, the accuracy of the model in each Alpha and Beta users are evaluated, separately. Given genuine and imposter as two states of the claimed identity, and considering Alpha and Beta users, four cases may occur in the authentication which are presented in Fig. 6. This figure shows the accuracy of each case separately. The results show that although the accuracy of the model in the case of Alpha users is slightly higher than the Beta users, in all four cases the model has more than $97\%$ accuracy, which shows the good performance of the model in extracting a universal fingerprint from EEG signals.

## Conclusion and future works

In this paper, we have focused on the practical challenges of EEG biometric authentication, including universality, privacy, and ease of use. For this aim, an EEG biometric authentication system was proposed which uses deep learning advantages to extract fingerprints of EEG signals. The system then verifies a user’s claimed identity by comparing the similarity between stored and presented EEG biometric fingerprints. Moreover, the proposed fingerprinting method, similar to the hash function, eliminates the possibility of accessing the EEG signals themselves and therefore protects user privacy as one of the major challenges here. Fingerprinting also has the universality capability and it can be used for all users, even those who are not in the model training data. For the aim of increasing user-friendliness and ease of use, in this paper, Gram-Schmidt procedure has been used to minimize the number of required channels while keeping the accuracy of the model. The case studies’ results showed that the proposed method is able to authenticate all users (including training and completely new users) using just three channels with more than $98\%$ accuracy which are outstanding results compared to previous works.

The challenges of privacy and universality in EEG biometric still need more attention and research. There exist methods that may improve these requirements. For example, siamese models, a deep learning-based tool for detecting identicality, may be used instead of the proposed fingerprinting method. Fuzzy hash functions, as another instance, which have been already used in many biometric factors to protect privacy, have not yet been employed in EEG biometric. In the future, we have planned to work on furthermore improving universality and privacy-preserving by examining more methods including the ones mentioned above.

## Nomenclatures

All the notations used in this manuscript, are defined in Table 5.

**Table 5:** Set of notations and their definitions.

**Signals**			
$u_k$	Raw signal of channel *k*-th	${\widehat{u}}_k$	Normalized signal of channel *k*-th
$v^i$	*i*-th orthogonal signal		
**Deep model and augmentation**			
*T*	Sliding window length	$\delta$	Sliding window step length
$\eta$	Number of moving of sliding window per input	$\Gamma$	Sampling window length
$\Delta$	Sampling window step length	*n*	Number of class or subjects
**Channel reduction**			
*E*	Set of available channels	*C*	Set of selected channels
*V*	Set of orthogonalized signals		
**Authentication Systems**			
*A*	set of Authentication information	*S*	set of Stored information
*f*	Complemntation function	*l*	Authentication function

## Data Availability

The datasets analysed during the current study are publicly available and accessible online at PhysioNet Database [https://physionet.org/content/eegmmidb/1.0.0/]^28,29^.
